# Comprehensive Expression Analysis of Cardiac Fibroblast Growth Factor 23 in Health and Pressure-induced Cardiac Hypertrophy

**DOI:** 10.3389/fcell.2021.791479

**Published:** 2022-01-18

**Authors:** Fiona Eitner, Beatrice Richter, Saskia Schwänen, Malgorzata Szaroszyk, Isabel Vogt, Andrea Grund, Thomas Thum, Joerg Heineke, Dieter Haffner, Maren Leifheit-Nestler

**Affiliations:** ^1^ Department of Pediatric Kidney, Liver and Metabolic Diseases, Pediatric Research Center, Hannover Medical School, Hannover, Germany; ^2^ Department for Cardiology and Angiology, Hannover Medical School, Hannover, Germany; ^3^ Institute of Molecular and Translational Therapeutic Strategies, Hannover Medical School, Hannover, Germany; ^4^ Department of Cardiovascular Physiology, European Center for Angioscience (ECAS), Medical Faculty Mannheim, University of Heidelberg, Mannheim, Germany

**Keywords:** FGF23, cardiac myocytes, cardiac fibroblasts, endothelial cells, left ventricular hypertrophy, fibrosis, transverse aortic constriction

## Abstract

Enhanced fibroblast growth factor 23 (FGF23) is associated with left ventricular hypertrophy (LVH) in patients with chronic kidney and heart disease. Experimentally, FGF23 directly induces cardiac hypertrophy and vice versa cardiac hypertrophy stimulates FGF23. Besides the bone, FGF23 is expressed by cardiac myocytes, whereas its synthesis in other cardiac cell types and its paracrine role in the heart in health and disease is unknown. By co-immunofluorescence staining of heart tissue of wild-type mice, we show that Fgf23 is expressed by cardiac myocytes, fibroblasts and endothelial cells. Cardiac Fgf23 mRNA and protein level increases from neonatal to six months of age, whereas no age-related changes in bone *Fgf23* mRNA expression were noted. Cardiac myocyte-specific disruption of *Fgf23* using Cre-LoxP system (Fgf23^fl/fl^/cre^+^) caused enhanced mortality, but no differences in cardiac function or structure. Although pressure overload-induced cardiac hypertrophy induced by transverse aortic constriction (TAC) resulted in a slightly worse phenotype with a more severe reduced ejection fraction, higher end-systolic volume and more enlarged systolic LV diameter in Fgf23^fl/fl^/cre^+^ mice compared to controls, this was not translated to any worse cellular hypertrophy, fibrosis or chamber remodeling. TAC induced *Fgf23* mRNA expression in whole cardiac tissue in both genotypes. Interestingly, co-immunofluorescence staining revealed enhanced Fgf23 synthesis in cardiac fibroblasts and endothelial cells but not in cardiac myocytes. RNA sequencing of isolated adult cardiac myocytes, cardiac fibroblasts and endothelial cells confirmed significantly higher *Fgf23* transcription in cardiac fibroblasts and endothelial cells after TAC. Our data indicate that Fgf23 is physiologically expressed in various cardiac cell types and that cardiac fibroblasts and endothelial cells might be an important source of FGF23 in pathological conditions. In addition, investigations in Fgf23^fl/fl^/cre^+^ mice suggest that cardiac myocyte-derived FGF23 is needed to maintain cardiac function during pressure overload.

## Introduction

Chronic kidney disease (CKD) is a worldwide health problem, and CKD patients suffer from an excessive high risk for cardiovascular disease (CVD) (Sarnak et al., 2003; Eckardt et al., 2013). Over 70% of patients with end-stage kidney disease (ESKD) develop pathological cardiac remodeling including hypertrophy and fibrosis, which promotes diastolic dysfunction, arrhythmia, heart failure and sudden death (Van Berlo et al., 2013; Ham et al., 2018). Hereby, the involvement of the bone-derived hormone fibroblast growth factor (FGF) 23 is widely discussed (Riminucci et al., 2003).

FGF23 synthesis in bone is induced in response to various stimuli to maintain phosphate homeostasis (Shimada et al., 2004; Shimada et al., 2005; Kawata et al., 2007; De Borst et al., 2011; Haussler et al., 2012; Rodriguez-Ortiz et al., 2012). Physiologically, FGF23 exerts its renal functions by binding to FGF receptor (FGFR) 1 in proximal tubular cells in the presence of its co-factor klotho causing intracellular activation of mitogen-activated protein kinase (MAPK) signaling leading to increased renal phosphate excretion and decreased 1,25-dihydroxy vitamin D synthesis (Shimada et al., 2004; Urakawa et al., 2006). CKD is a state of high phosphate load and therefore FGF23 levels in the circulation already start to rise in early stages of CKD and reach a 1,000-fold increase in ESKD (Weber et al., 2003; Gutierrez et al., 2005). In parallel, the prevalence of left ventricular hypertrophy (LVH) increases and is associated with a higher mortality rate in these patients (Go et al., 2004; Paoletti et al., 2016). Furthermore, increased circulating FGF23 in CKD is linked to LVH development and mortality (Faul et al., 2011). *In vitro* and *in vivo* data show that FGF23 induces LVH by directly targeting the heart *via* binding and phosphorylating the intracellular receptor kinase domain of FGFR4 leading to the activation of the pro-hypertrophic calcineurin/nuclear factor of activated T cells (NFAT) signaling pathway in a klotho-independent manner (Faul et al., 2011; Grabner et al., 2015). Interestingly, studies in the general population have shown that elevated FGF23 levels are also associated with CVD and mortality independent of the presence of CKD (Kestenbaum et al., 2014; Lutsey et al., 2014). Furthermore, FGF23 promotes myocardial fibrosis *via* activation of β-Catenin (Hao et al., 2016), activates the intra-cardiac renin-angiotensin-aldosterone system (RAAS), which in turn promotes LVH and cardiac fibrosis (Böckmann et al., 2019), and it is associated with endothelial dysfunction in CKD patients (Yilmaz et al., 2010), and in human coronary artery endothelial cells *in vitro* (Richter et al., 2016).

FGF23 expression is not limited to the bone since its synthesis has been also found in the heart in cardiac myocytes (Fon Tacer et al., 2010; Andrukhova et al., 2015; Hao et al., 2016; Leifheit-Nestler et al., 2016; Kuga et al., 2020) and in human coronary arteries in patients with impaired kidney function (Van Venrooij et al., 2014). However, its expression in non-myocytes is still controversially discussed (Schumacher et al., 2019). Likewise, the impact of cardiac-derived FGF23 and its possible paracrine actions in the heart in comparison to endocrine-acting FGF23 secreted from bone in health and disease are largely elusive (Leifheit-Nestler et al., 2018).

Here, we show that Fgf23 is expressed in the left as well as the right ventricle and in the respective atria in healthy wild-type mice. In addition to cardiac myocytes, cardiac fibroblasts and endothelial cells synthesize Fgf23 too. To more finely examine the function of cardiac-derived Fgf23, we generated conditional Fgf23 knockout mice by cross-breeding mice with LoxP-flanked (floxed) *Fgf23* alleles with mice harboring a cardiac myocyte-specific recombination. Cardiac myocyte-specific *Fgf23* knockout mice showed normal cardiac phenotype under basic conditions, but exhibited a slightly worse cardiac function after transverse aortic constriction (TAC) in comparison to TAC-operated controls that however, was not translated to any worse cellular hypertrophy, fibrosis or chamber remodeling. Interestingly, cardiac myocyte-specific *Fgf23* knockout mice showed elevated *Fgf23* expression in total cardiac tissue after TAC, raising the question whether cardiac fibroblasts and endothelial cells might be an important source of FGF23 in pathological conditions.

## Materials and Methods

### Generation of Conditional Cardiac Myocyte-specific *Fgf23* Knockout Mice

Animal experiments were approved by the State Office Committee for Animal Welfare Lower Saxony (LAVES, #15/1912) in accordance with national animal protection guidelines from Directive 2016/63/EU of the European Parliament on the protection of animals used for scientific purposes. Mice were housed with a 12:12 h light-dark cycle, had free access to water and fed *ad libitum* with standard rodent chow. The Myh7-Cre mouse line expressing Cre recombinase linked to a Myh7 promotor (B6;129S1-Tg(Myh7-Cre)1Jmk) was kindly provided by Prof. Joerg Heineke (Department of Cardiovascular Physiology, European Center for Angioscience (ECAS), Medical Faculty Mannheim, University of Heidelberg, Mannheim, Germany) and described elsewhere (Heineke et al., 2007). Fgf23 floxed mice were generated based on CRISPR/Cas9 technology by InGenious Targeting, Inc. (Ronkonkoma, NY, United States). To obtain mice with cardiac myocyte-specific *Fgf23* deletion, homozygous Fgf23^fl/fl^ mice (B6-Fgf23^tm1Mln^) were interbred with B6;129S1-Tg(Myh7-Cre)1^Jmk^. Fgf23^fl/fl^/cre- littermates were used as controls.

For genotyping, earmarks were lysed in proteinase K buffer overnight. After deactivation of proteinase K, multiplex PCR was performed to amplify genomic DNA using primer for Fgf23 floxed allele (forward: AGA TTC CAT TTA CAG TGC CCC TTG G; reverse: ACT TCA GTT ACC TGA AGT CCC AGT TGG) and Myh7-Cre (forward: GGC GTT TTC TGA GCA TAC CT; reverse: CTA CAC CAG AGA CGG AAA TCC). PCR was run for 3 min at 94°C and 31 cycles of 30 s at 94°C, 45 s at 58°C, and 1 min at 72°C, followed by 10 min at 72°C. Samples were separate on a 3% agarose gel including GelRed Nucleic Acid Gel Stain (Biotrend) at 120 V for 60 min and analyzed with a Gel DocTMXR+ (Bio-Rad Laboratories).

Respective male and female Fgf23^fl/fl^/cre^−^ and Fgf23^fl/fl^/cre^+^ mice with the age of 0 (neonatal), 1, 3 and 6 months were analyzed. Femur, tibia, and calvaria were isolated and snap-frozen in liquid nitrogen. Isolated mouse hearts were washed in ice-cold PBS followed by 0.5% potassium chloride and cut into three cross-sections. For histological evaluation, the mid cross-section was fixated in 4% RotiHistofix (Carl Roth) and embedded in paraffin. The other two cross-sections were snap-frozen in liquid nitrogen and stored at −80°C for molecular and biochemical analyses.

### Transthoracic Echocardiography and Cardiac Catheterization

Echocardiography was performed using the Vevo 2100 Imaging System (FUJIFILM VisualSonics Inc.) as described previously (Zacchigna et al., 2021). Anesthesia was induced with 3% isoflurane and maintained with 0.5–1% isoflurane over a breathing mask. Heart views were acquired in parasternal long axis in B- and M-mode, and short axis in M-mode. All echocardiographic images were analyzed using the Vevo Lab 3.2.0 software.

Cardiac catheterization was performed with PowerLab 16/35 (Millar Instruments). Anesthesia was initialized with 3% isoflurane and maintained with 0.8% isoflurane. The measurements were performed on the opened thorax using the polyamide catheter PVR 1045 (Millar Instruments) and analyzed with the software LabChart 8 (AD Instruments).

### Transverse Aortic Constriction

Transverse aortic constriction (TAC) or sham surgery was performed in eight-week-old male and female Fgf23^fl/fl^/cre^−^ and Fgf23^fl/fl^/cre^+^ mice by subjecting the aorta to a defined constriction as previously described (Grund et al., 2019a). In brief, a 7–0 silk suture was tied around the aortic arch between the branches of the brachiocephalic trunk and the left common carotid artery and a blunted 27-gauge needle, after which the needle was removed to create a defined constriction. The procedure for sham-operated animals was identical, but the aorta was not ligated. Two weeks after surgery, mice were sacrificed as described above.

Cardiac specific cell types were isolated from mice after sham and TAC as previously described (Appari et al., 2017). In brief, ventricular cardiac myocytes were isolated using a Langendorff system, cardiac endothelial cells and fibroblasts were isolated using MACS technology with CD146 microbeads followed by feeder removal microbeads (Miltenyi Biotec). All isolated cell types were directly used for RNA extraction according to standard protocol followed by deep-sequencing analysis as previously described (Grund et al., 2019b).

### Cardiac Magnetic Resonance Imaging

Cardiac magnetic resonance imaging (MRI) was performed for TAC or sham-operated Fgf23^fl/fl^/cre^−^ and Fgf23^fl/fl^/cre^+^ mice. Cardiac MRI images were recorded with a Bruker 7T Pharmascan 70/16 (Bruker). Anesthesia was induced with 3% isoflurane and maintained with 0.5% isoflurane. First, a positioning scan was performed, followed by images in 9–10 stacks. For every stack, images were made to encompass an entire heart cycle. All cardiac MRI images were analyzed with Mass4Mice 7.1 software (MEDIS Medical Technologies). Epicardium and endocardium were marked manually to ascertain left ventricular (LV) mass, end-systolic (ES) and end-diastolic (ED) volume, stroke volume (SV), cardiac output, and ejection fraction (EF). In addition, LV wall geometry was determined at the level of papillary muscles. With regard to slice thickness, papillary muscles were included in the LV cavity.

### Blood and Urine Biochemistry

Plasma was analyzed for total FGF23 (#60-6300) and intact FGF23 (#60-6800) by ELISA (both from Immutopics). Urine and serum biochemistry were measured on a cobas c111 system (Roche Diagnostics) using Calcium Gen.2, Phosphate ver.2 and creatinine Creatinine Jaffé Gen.2 (all from Roche Diagnostics). No urine was available for one month old Fgf23^fl/fl^/cre^+^ and Fgf23^fl/fl^/cre^−^ mice.

### Histological Staining of Heart Tissue and Quantification of Cardiac Myocyte Size, Capillarization and LV Fibrosis

Three µm thick cardiac mid-chamber sections were deparaffinized in xylene and hydrated using a descending alcohol series. Hematoxylin and eosin (HE) staining was performed using Meyers hematoxylin and eosin G (Merck Millipore). To visualize interstitial cardiac fibrosis, sections were incubated using picrosirius red solution (Merck Millipore) in 1.2% picric acid. For immunohistochemical staining of FGF23 in heart tissue, antigen retrieval was done in citrate buffer pH 6.0, followed by inhibition of endogenous peroxidase activity by hydrogen peroxidase and blocking with 10% normal goat serum in PBS. After incubation with anti-FGF23 primary antibody (Bioss #bs-5768R, dilution 1:200), sections were incubated with horseradish peroxidase-conjugated goat anti-rabbit (Jackson ImmunoResearch #111-035-144, dilution 1:100), followed by incubation with diaminobenzidine substrate (Thermo Scientific #34065) and counterstaining with hematoxylin. Images were taken with either 10x (HE) or 20x objective using the Axio Observer Z1 microscope (Carl Zeiss). To quantify interstitial cardiac fibrosis, five pictures per mouse of the left ventricle were taken using 20x magnification followed by quantification using ImageJ (Böckmann et al., 2019).

For visualization of cell type-specific expression of FGF23, cardiac tissue was stained for anti-CD31 (R&D systems #AF3628; dilution 1:100 in 5% milk in TBST), followed by donkey-anti-goat Alexa Fluor 480 secondary antibody (Invitrogen #A11055, dilution 1:500), anti-α-Actinin (Sigma-Aldrich #A7732, dilution 1:100), followed by goat-anti-mouse Alexa Fluor 660 secondary antibody (Invitrogen #A21054, dilution 1:500), or anti-PDGFRα (R&D Systems #AF1062, dilution 1:100), followed by donkey-anti-goat Alexa Fluor 480 secondary antibody. Afterwards, tissues were stained with anti-FGF23 (Bioss #bs-5768R, dilution 1:200), followed by goat-anti-rabbit Alexa Fluor 555 secondary antibody (Invitrogen #A21428, dilution 1:500). All primary antibodies were incubated over night at 4°C, and secondary antibodies were incubated for 90 min at room temperature. For measurement of capillarization and cardiac myocyte size, the staining with CD31 was followed by incubation with 5 µg/ml wheat germ agglutinin (WGA)Alexa Fluor 555 (Invitrogen #W32464) for one hour. Nuclear counterstaining in immunofluorescence was performed with DAPI. All images were taken with a 20x objective on a Zeiss Axio Observer Z1 microscope (Carl Zeiss) and quantification of cardiac myocyte area was performed with 100 cells per animal using the Zen 2.3 lite software (Carl Zeiss). For quantification of capillarization, five images in 20x magnification per animal were taken and number of capillaries were divided by number of cardiac myocytes per power field.

### RNA Isolation, cDNA Synthesis and Quantitative Real-Time PCR Analysis

**FIGURE 1:**
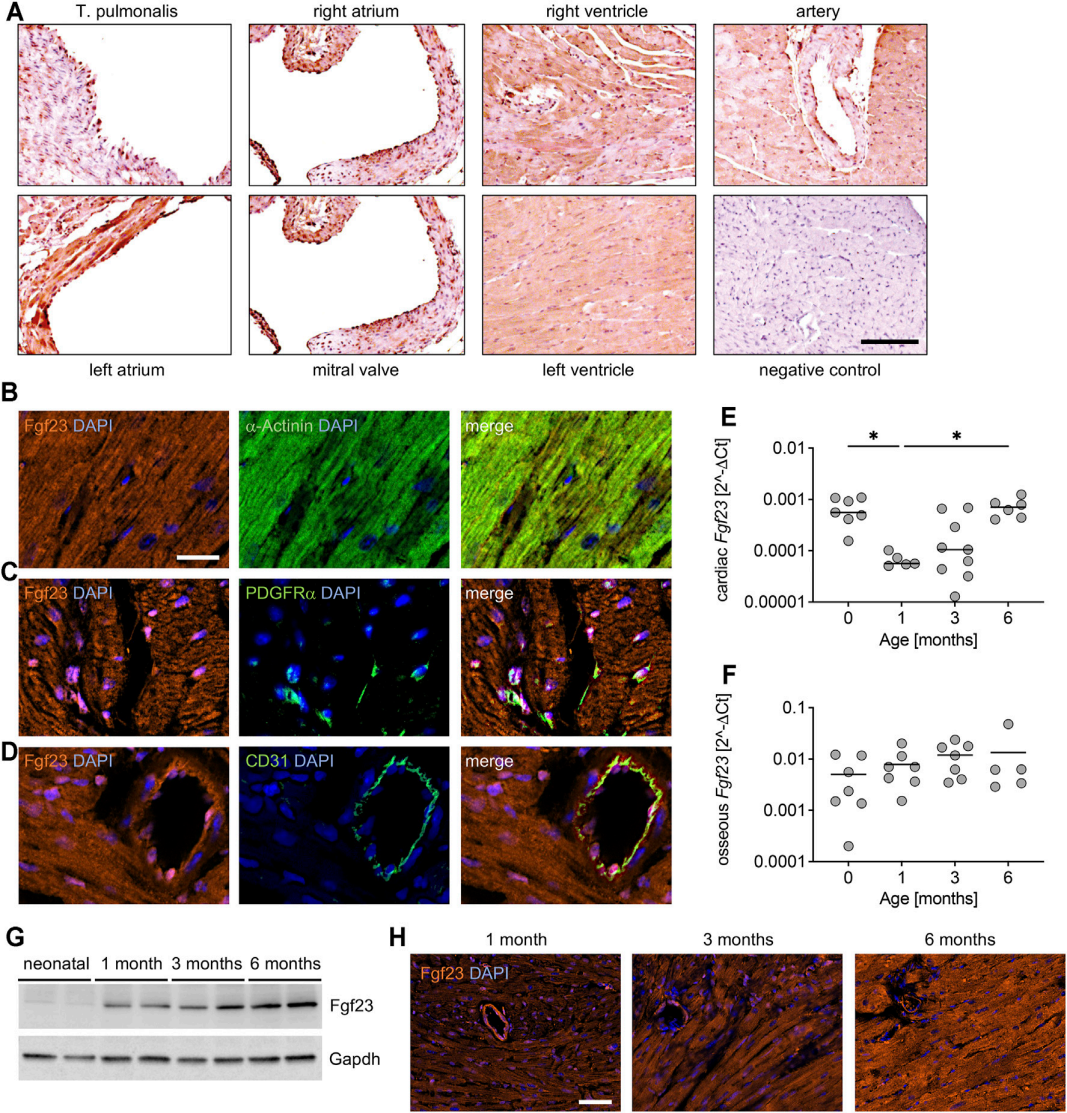
Expression pattern of Fgf23 in healthy murine heart tissue. **(A)** Representative immunohistochemical staining for Fgf23 (brown color) in hearts tissue of adult C57BL/6N mice. Original magnification ×10, scale bar 100 µm. **(B–D)** Representative immunofluorescence co-staining in cardiac tissue from adult C57BL/6N mice for Fgf23 (orange) and **(B)** the cardiac myocyte-specific marker α-Actinin, **(C)** the fibroblast-specific marker platelet-derived growth factor receptor (PDGFR) α, and **(D)** the endothelial-specific marker CD31 (each in green). DAPI (blue) is used for nuclear counterstaining. Magnification ×20, scale bar 20 µm. **(E, F)** Relative *Fgf23* mRNA expression in **(E)** heart and **(F)** bone of neonatal to 6 months old C57BL/6N mice analyzed by quantitative real-time PCR. *Gapdh* is used as housekeeping gene to calculate 2^−ΔCt^ values. **(G)** Representative immunoblot of Fgf23 protein in total heart tissue lysates of neonatal to 6 months old C57BL/6N mice. Gapdh serves as loading control. **(H)** Age-dependent immunofluorescence staining for Fgf23 (orange) in cardiac mid-chamber cross-sections of 1–6 months old C57BL/6N mice. Nuclei are counterstained with DAPI (blue). Magnification ×20, scale bar 50 µm. Data is given as scatter dot plots with means; *p < 0.05 analyzed using Kruskal-Wallis test followed by Dunn’s multiple comparison test according to Shapiro-Wilk normality test; *n* = 5–9 mice per group.

**FIGURE 2:**
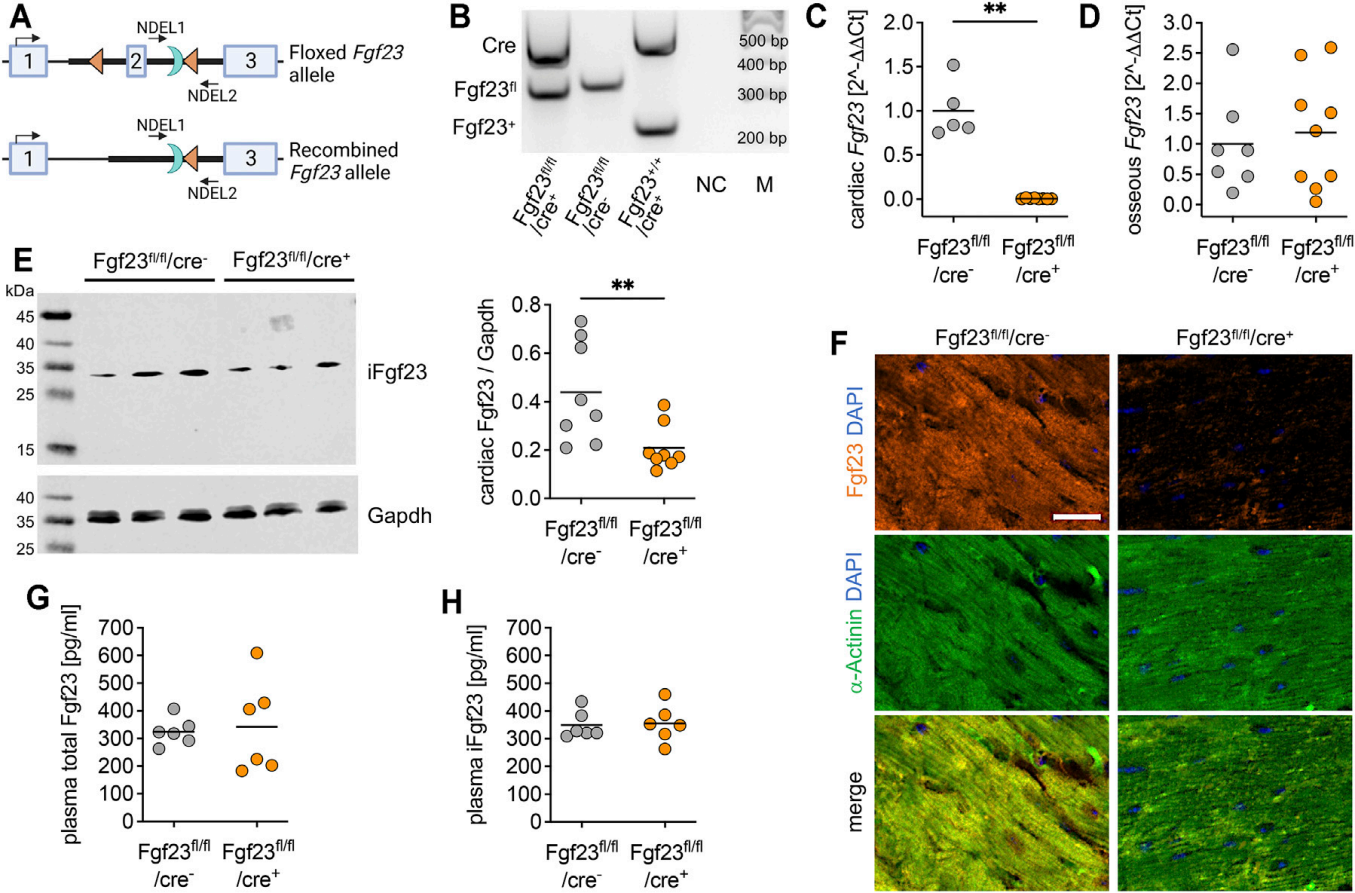
Characterization of the cardiac myocyte-specific Fgf23 knockout mouse. **(A)** Schematic illustration of CreLoxP strategy to generate cardiac myocyte-specific Fgf23 knockout showing murine *Fgf23* allele with exons 1-3 and location of LoxP sites (triangles) surrounding exon 2 as well as the remaining Flp recognition target site (half-moon). Positions of PCR primer for genotyping are marked with NDEL1 and 2. Top: floxed *Fgf23* allele, bottom: *Fgf23* allele after Cre-mediated recombination (generated with BioRender.com). **(B)** Representative agarose gel of genotyping PCR demonstrating PCR products for wild-type (Fgf23^+^; 253 bp) or floxed (Fgf23^fl^, 367 bp) *Fgf23* allele and Cre (550 bp), a non-template control (NC), and 100-bp DNA ladder (M). **(C, D)** Quantitative real-time PCR analysis reveals almost undetectable cardiac, but unchanged osseous *Fgf23* mRNA in Fgf23^fl/fl^/cre^+^ compared to Fgf23^fl/fl^/cre^−^ control mice. **(E)** Representative immunoblot and respective quantification show significantly reduced Fgf23 protein levels in total heart lysates of Fgf23^fl/fl^/cre^+^ compared to Fgf23^fl/fl^/cre^−^ control mice. Gapdh serves as loading control. **(F)** Immunofluorescence co-staining for Fgf23 (orange) and α-Actinin (green) with cell nuclei (blue) demonstrates lacking Fgf23 protein expression in cardiac myocytes of Fgf23^fl/fl^/cre^+^ mice compared to Fgf23^fl/fl^/cre^−^ controls. Magnification ×20, scale bar 20 µm. **(G)** Plasma total Fg23 and **(H)** intact Fgf23 (iFgf23) concentrations in Fgf23^fl/fl^/cre^+^ mice are comparable with respective Fgf23^fl/fl^/cre^−^ controls. Data is given as scatter dot plots with means; **p < 0.01 analyzed using unpaired *t*-tests according to Shapiro-Wilk normality test; *n* = 5–9 mice per group.

The RNeasy Mini Kit was used according to manufacturer’s protocol for total RNA isolation from murine heart and cardiac specific cell types. For murine bone (either calvaria or femur), the RNeasy Lipid Tissue Kit was used. 500 ng RNA was transcribed into cDNA according to the data sheet of the QuantiTect Reverse Transcription Kit. Real-time polymerase chain reaction (PCR) was performed in triplets using the QuantiFAST SYBR Green PCR Kit (all kits from Qiagen) on a 7900 HT Fast RT-PCR System (Thermo Fisher Scientific) and analyzed using the SDS Software v2.4 (Thermo Fisher Scientific). For murine primer sequences see Supplementary Table S1. Relative gene expression levels of Fgf23^fl/fl^/cre^+^ mice compared to control were calculated using the 2^−ΔΔCt^ method, with *Gapdh* and *18s* (in bone) serving as housekeeping genes. For *Fgf23* mRNA quantification in Figures 1, 4, 2^−ΔCt^ values were used.

### Protein Isolation and Western Blot Analysis

Protein isolation of murine heart tissue was performed with the TissueLyser LT (Qiagen) using RIPA buffer with proteinase and phosphatase inhibitors (Sigma-Aldrich). Samples were incubated at 4°C for 30 min followed by sonification. Protein concentrations were measured *via* BCA assay (Thermo Fisher Scientific) according to manufacturer’s protocol.

100 µg total protein was separated on a 12% SDS-gel and transferred on nitrocellulose membrane in transfer buffer containing 18% methanol for 1 h. Membranes were blocked in 5% milk in TBS containing 0.05% Tween-20 for 1 h, afterwards primary antibodies were incubated over-night at 4°C (FGF23-6310 Goat mAb, dilution 1:500, Immutopics; GAPDH 14C10 Rabbit mAb, dilution 1:1,000, Cell Signaling Technology). After washing, secondary antibodies were incubated for 1 h at room temperature (Goat anti-rabbit IgG HRP, dilution 1:1,000, Santa Cruz; Donkey anti-goat IgG HRP, dilution 1:2000, R and D Systems). ECL development was done with the SuperSignal West Femto Maximum Sensitivity Substrate (Thermo Fisher Scientific) and signals were detected with the Odyssey FC Imaging System (LI-COR Bioscience). Expression levels were quantified with the Image Studio Lite 5.2 software (LI-COR Bioscience).

### Statistical Analyses

All statistical analyses were performed using GraphPad Prism software 7 (GraphPad Software) Gaussian’s distribution was analyzed by Shapiro-Wilk test. Differences between two groups were determined using 2-tailed Students *t*-test or Mann Whitney *U* test, respectively. For time-dependent analyses, one-way ANOVA or Kruskal-Wallis test followed by Dunnett or Dunn’s multiple comparison tests were used. TAC-experiments were analyzed by two-way ANOVA followed by Tukey’s multiple comparison test. *p* values < 0.05 were considered as statistically significant.

## Results

### The Healthy Murine Heart Is a Source of Fgf23

Most clinical and experimental studies over the past decade focused on FGF23 in the context of LV pathologies (Faul et al., 2011; Grabner et al., 2015; Matsui et al., 2018). It has not been investigated so far in which areas of the whole heart Fgf23 is locally expressed. Therefore, we first performed immunohistochemical staining for Fgf23 using longitudinal sections of healthy adult C57BL/6N wild-type mice. The expression of Fgf23 was comparable between the left and the right ventricle (Figure 1A). In direct comparison, the synthesis of Fgf23 was higher in the left atrium than in the right atrium (Figure 1A). Fgf23 was clearly expressed in all regions of endocardium and endothelium (Figure 1A), while it appeared to be only minimal in the *tunica media* of the depicted vessels, which is predominantly composed of vascular smooth muscle cells (Figure 1A). To further elucidate the expression pattern of murine Fgf23 within specific cardiac cell types, we performed co-immunofluorescence staining of Fgf23 with cell type specific markers for cardiac myocytes, fibroblasts and endothelial cells in formalin-fixed paraffin-embedded cardiac cross-sections of adult C57BL/6N wild-type mice. Fgf23 was co-localized with the cardiac myocyte-specific marker α-Actinin, verifying, that healthy cardiac myocytes express Fgf23 (Figure 1B). Fgf23 was further expressed in platelet-derived growth factor receptor A (PDGFRα) positive cardiac fibroblast (Figure 1C) and co-localized with CD31, known as platelet endothelial cell adhesion molecule-1 (PECAM-1), suggesting the expression of Fgf23 in endothelial cells of the murine heart, too (Figure 1D).

Studies in humans showed that circulating total FGF23 is highest in infants and adolescents and lowest at adult age (Fischer et al., 2012), but the age-dependent synthesis of FGF23 in the heart and bone has not been studied so far. Therefore, we next investigated the Fgf23 expression in heart and bone tissue of C57BL/6N mice from neonatal (0) to 6 months of age. Although the individual variability of mice was high, cardiac *Fgf23* mRNA levels were significantly lower in 1 month old mice compared to neonates and increased again with increasing age (Figure 1E), whereas no age-related changes in bone *Fgf23* mRNA expression were noted (Figure 1F). Of note, osseous expression of *Fgf23* was approximately 20-fold higher than in the heart. Immunoblot analysis of total cardiac tissue confirmed an age-dependent increase of Fgf23 protein synthesis (Figure 1G), which was verified by immunofluorescence staining of Fgf23 in cardiac mid-chamber cross-sections (Figure 1H). It appears that the increasing total amount of cardiac Fgf23 originates mainly from cardiac myocytes and that expression in endothelial cells and fibroblasts remains approximately the same with increasing age.

Taken together, Fgf23 is not only expressed in cardiac myocytes, but also in cardiac fibroblasts and endothelial cells, suggesting that the heart is a source of Fgf23.

### Mice With Cardiac Myocyte-specific Fgf23 Deletion Reveal Normal Circulating Fgf23 Levels

The physiological role of bone-derived endocrine-acting FGF23 in renal phosphate homeostasis is well-established (Shimada et al., 2004), whereas the role of intra-cardiac FGF23 synthesis has been poorly studied. To investigate cardiac-derived FGF23 in more detail, we generated a cardiac myocyte-specific Fgf23 knockout mouse using Cre-LoxP system (Figure 2A). The first LoxP site was inserted 222 bp upstream of exon 2 and the Neo cassette, containing the second LoxP site, was inserted 342 bp downstream of exon 2. The size of the target region was 668 bp containing exon 2. The respective Fgf23-LoxP construct was delivered to iTL BF1 (C57BL/6 FLP) embryonic stem cells, which were microinjected into Balb/c blastocysts using standard protocols. Resulting chimeras with high percentage black fur color were mated to C57BL/6 wild-type mice to generate germline floxed Fgf23 mice. After intercrossing Fgf23^fl/+^ mice, homozygous non-recombined Fgf23-LoxP mice (Fgf23^fl/fl^) were mated with transgenic cardiac myocyte-specific B6;129S1-Tg(Myh7-Cre)^1Jmk^ mice by standard breeding regimen. As published elsewhere, in B6;129S1-Tg(Myh7-Cre)^1Jmk^ mice, the Cre enzyme has a significant activity at embryonic day 17.5 (Oka et al., 2006). Finally, female Fgf23^fl/fl^/Myh7-cre^−^ mice were bred with males fully recombined for *Fgf23* (Fgf23^fl/fl^/Myh7-cre^+^). To verify genotypes, PCR products were amplified from genomic DNA using primer pairs spanning the floxed exon 2 region of murine *Fgf23* and Cre recombinase in a semi-quantitative multiplex PCR (see Methods). Thereby a 253 bp product represented the wild-type *Fgf23* allele, a 367 bp product the floxed *Fgf23* allele, and a 550 bp product the *Cre* expression (Figure 2B). Male and female Fgf23^fl/fl^/Myh7-cre^+^ mice (in the following termed as Fgf23^fl/fl^/cre^+^) were analyzed in all experiments and respective non-recombined Fgf23^fl/fl^/Myh7-cre^−^ littermates (in the following termed as Fgf23^fl/fl^/cre^−^) were used as controls. First, we verified the cardiac myocyte-specific *Fgf23* knockout. Thereby, Fgf23^fl/fl^/cre^+^ mice showed significantly reduced cardiac *Fgf23* mRNA expression compared to controls (Figure 2C), while their osseous *Fgf23* synthesis remained unaffected (Figure 2D). Western blot analysis showed significantly reduced full-length cardiac Fgf23 protein in Fgf23^fl/fl^/cre^+^ compared to control mice (Figure 2E). Using anti-α-Actinin antibody, immunofluorescence microscopy confirmed the cardiac myocytes-specific *Fgf23* knockout (Figure 2F). However, circulating total and intact Fgf23 (iFgf23) levels in Fgf23^fl/fl^/cre^+^ mice were comparable to controls (Figures 2G,H), and phosphate and calcium metabolism remained unchanged up to six months of age (Supplementary Table S2).

### Cardiac Myocyte-specific Fgf23 Deletion Does Not Affect Cardiac Function or Structure in Unchallenged Mice

**FIGURE 3:**
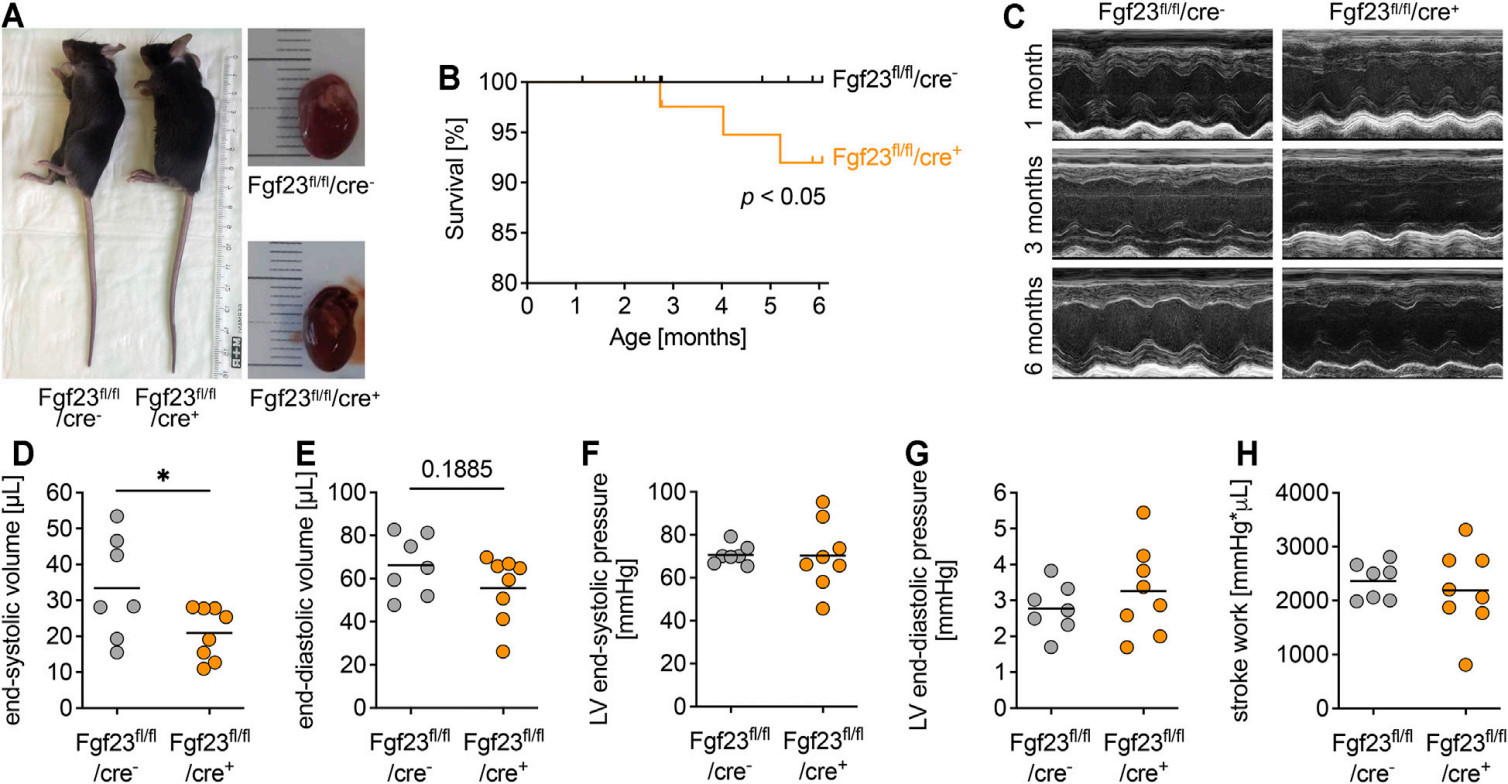
Cardiac-myocyte specific *Fgf23* knockout mice do not display changes in cardiac function and geometry. **(A)** Body length and heart size are not different between three months old Fgf23^fl/fl^/cre^+^ and Fgf23^fl/fl^/cre^−^ control mice. **(B)** Fgf23^fl/fl^/cre^+^ mice have an increased mortality rate within six months of age. *p < 0.05 analyzed using Log-rank test; *n* = 54–79 mice per genotype. **(C)** Representative parasternal long-axis M-mode echocardiography images of 1, 3 and 6 months old Fgf23^fl/fl^/cre^−^ and Fgf23^fl/fl^/cre^+^ mice. **(D**–**H)** Quantification of end-systolic and end-diastolic volume, end-systolic and end-diastolic pressure, and stroke work using echocardiography and Millar catheter, respectively, in six months old Fgf23^fl/fl^/cre^−^ and Fgf23^fl/fl^/cre^+^ mice. Data is given as scatter dot plots with means; *p < 0.05 analyzed using unpaired *t*-tests according to Shapiro-Wilk normality test; *n* = 7–8 mice per group.

Mice with cardiac myocyte specific Fgf23 deletion showed normal body length and heart size compared to age-matched controls (Figure 3A). The body weight related organ weights including heart, lung, kidney and liver increased with age, but did not differ between both genotypes (Supplementary Table S3). However, the mortality of Fgf23^fl/fl^/cre^+^ mice during the first 6 months of life was slightly but significantly increased compared to controls (Figure 3B). Next, we performed echocardiography and Millar catheter to determine whether cardiac function was affected in Fgf23^fl/fl^/cre^+^ mice, which in turn may promote their increased mortality. In echocardiography, no differences between Fgf23^fl/fl^/cre^−^ and Fgf23^fl/fl^/cre^+^ mice were observed, irrespective of age (Figure 3C, Supplementary Table S4), except for a significantly reduced end-systolic volume (ESV) and slightly lower end-diastolic volume (EDV) in six months old Fgf23^fl/fl^/cre^+^ mice compared to control (Figures 3D,E). Using Millar catheter, no differences were found in end-systolic or end-diastolic LV pressure or stroke work (Figures 3F,G). Overall, these data indicate no pathologic cardiac phenotype in Fgf23^fl/fl^/cre^+^ mice.

**FIGURE 4:**
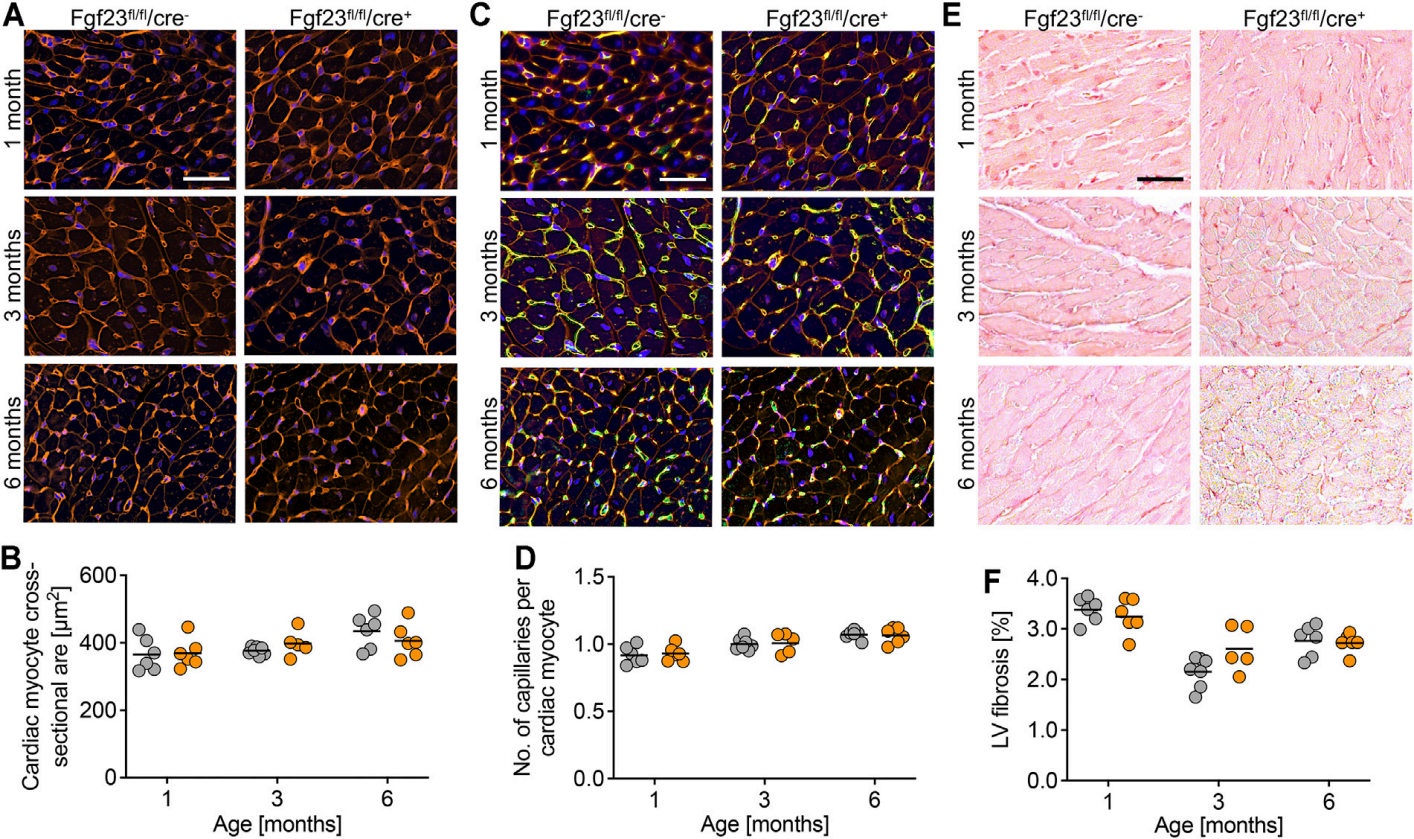
Cardiac myocyte-specific Fgf23 knockout mice do not show cellular and molecular changes in the heart. **(A)** Immunofluorescence staining of heart tissue from 1, 3 and 6 months old Fgf23^fl/fl^/cre^−^ and Fgf23^fl/fl^/cre^+^ mice with wheat germ agglutinin (WGA) Alexa Fluor 555 (red) and nuclei (blue). Magnification ×20, scale bar 50 µm. **(B)** Quantification of cardiac myocyte cross-sectional area does not show differences between both genotypes. **(C)** Immunofluorescence co-staining for WGA (red), CD31 (green) and DAPI (blue) in heart tissue from 1, 3 and 6 months old Fgf23^fl/fl^/cre^−^ and Fgf23^fl/fl^/cre^+^ mice. Magnification ×20, scale bar 50 µm. **(D)** Quantification of capillary number per cardiac myocyte does not differ between both genotypes. **(E)** Picrosirius red staining in heart tissue from 1, 3 and 6 months old Fgf23^fl/fl^/cre^−^ and Fgf23^fl/fl^/cre^+^ mice. Magnification ×10, scale bar 50 µm. **(F)** Cardiac myocyte-specific *Fgf23* deletion does not induce left ventricular (LV) fibrosis, although differences over time are observed. Data is given as scatter dot plots with means analyzed by Two-way ANOVA followed by Tukey’s multiple comparison test; *n* = 5–7 mice per group.

Next, we performed histological analyses to determine alterations in the cardiac structure of Fgf23^fl/fl^/cre^+^ and control mice. Quantified by wheat germ agglutinin (WGA) staining and fluorescence microscopy, cardiac myocyte cross-sectional area did not differ between the genotypes (Figures 4A,B). This was in line with the equal expression of the pro-hypertrophic markers atrial natriuretic peptide (*ANP*), brain natriuretic peptide (*BNP*) and beta myosin heavy chain (*bMHC*) in Fgf23^fl/fl^/cre^+^ mice compared to controls, irrespective of age (Supplementary Figures S1A–C). Neither the capillary density (Figures 4C,D) nor the accumulation of interstitial collagen fibers (Figures 4E,F) were affected by the conditional cell-specific disruption of *Fgf23* in cardiac myocytes over time. The mRNA expression of the pro-fibrotic marker transforming growth factor beta 1 (*Tgfb1*) and its downstream target connective tissue growth factor (*Ctgf*) was equal between both groups (Supplementary Figures S1D,E). Consistently, collagen type 1, alpha 1, recently identified as a marker in human heart failure progression related to tissue fibrosis (Hua et al., 2020) and encoded by the gene *Col1a1*, was not differentially expressed in Fgf23^fl/fl^/cre^+^ mice compared to age-matched controls (Supplementary Figure S1F). Thus, in line with normal heart function in echocardiography and Millar catheter, conditional deletion of *Fgf23* in cardiac myocytes does not alter cardiac structure.

### Loss of Cardiac Myocyte-specific Fgf23 Does Not Alter Impaired Cardiac Function After Transverse Aortic Constriction

**FIGURE 5:**
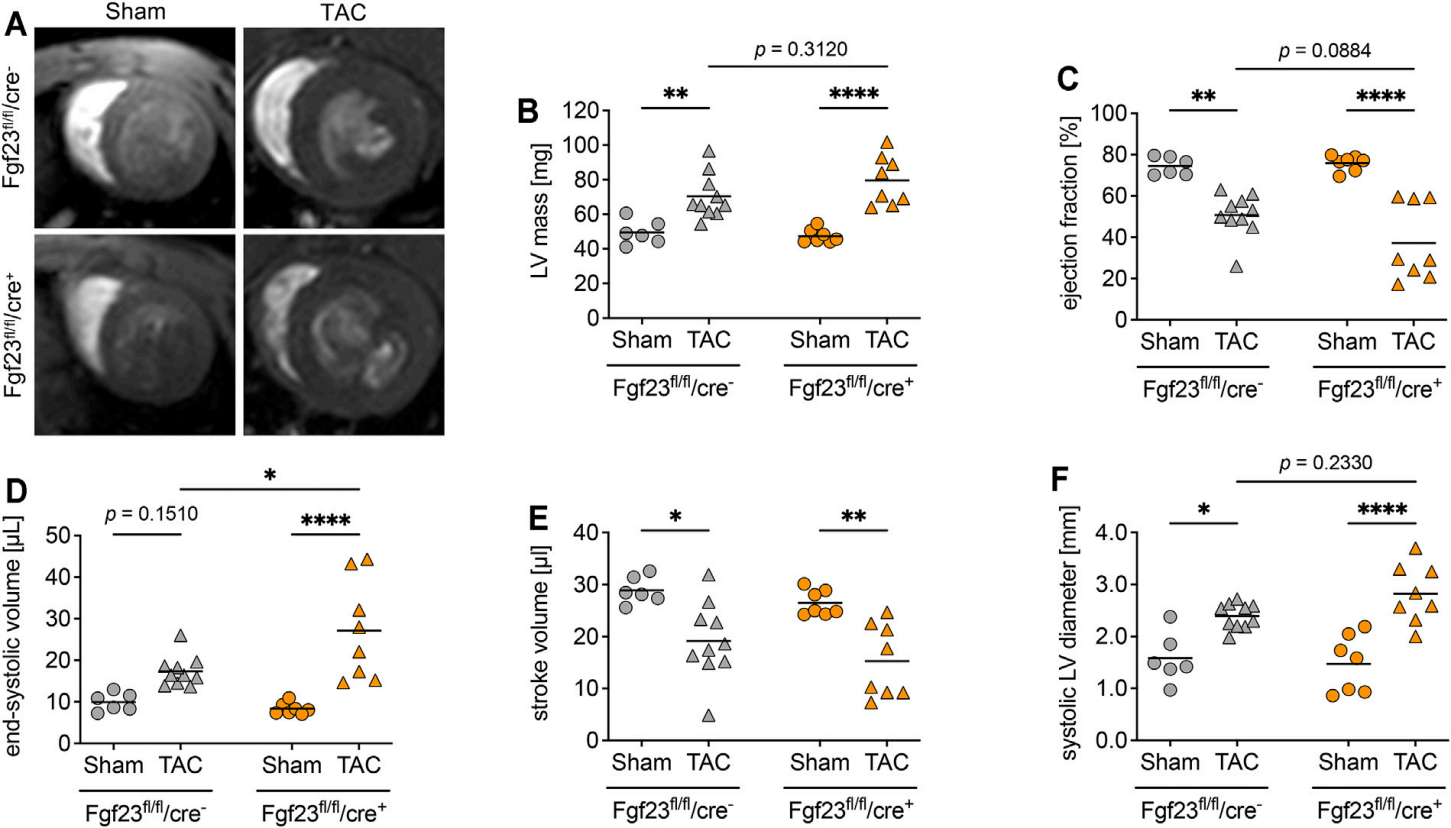
Cardiac myocyte-specific *Fgf23* knockout worsen high blood pressure-induced cardiac dysfunction. **(A)** Representative cardiac cross-sectional magnetic resonance images of sham and TAC-operated Fgf23^fl/fl^/cre^−^ and Fgf23^fl/fl^/cre^+^ mice. **(B)** Left ventricular (LV) mass is enhanced in TAC-operated Fgf23^fl/fl^/cre^−^ and Fgf23^fl/fl^/cre^+^ mice. **(C)** Ejection fraction is reduced after TAC surgery, which is more pronounced in Fgf23^fl/fl^/cre^+^ mice. **(D)** TAC increases the end-systolic volume (ESV) in both genotypes, with ESV being significantly higher in Fgf23^fl/fl^/cre^+^ mice compared to controls after TAC. **(E)** Stroke volume is reduced in both genotypes after TAC. **(F)** Systolic LV diameter is enhanced after TAC, which is more pronounced in Fgf23^fl/fl^/cre^+^ mice. Data is given as scatter dot plots with means; *p < 0.05, **p < 0.01, and ****p < 0.0001 analyzed by Two-way ANOVA followed by Tukey’s multiple comparison test; *n* = 6–10 mice per group.

**FIGURE 6:**
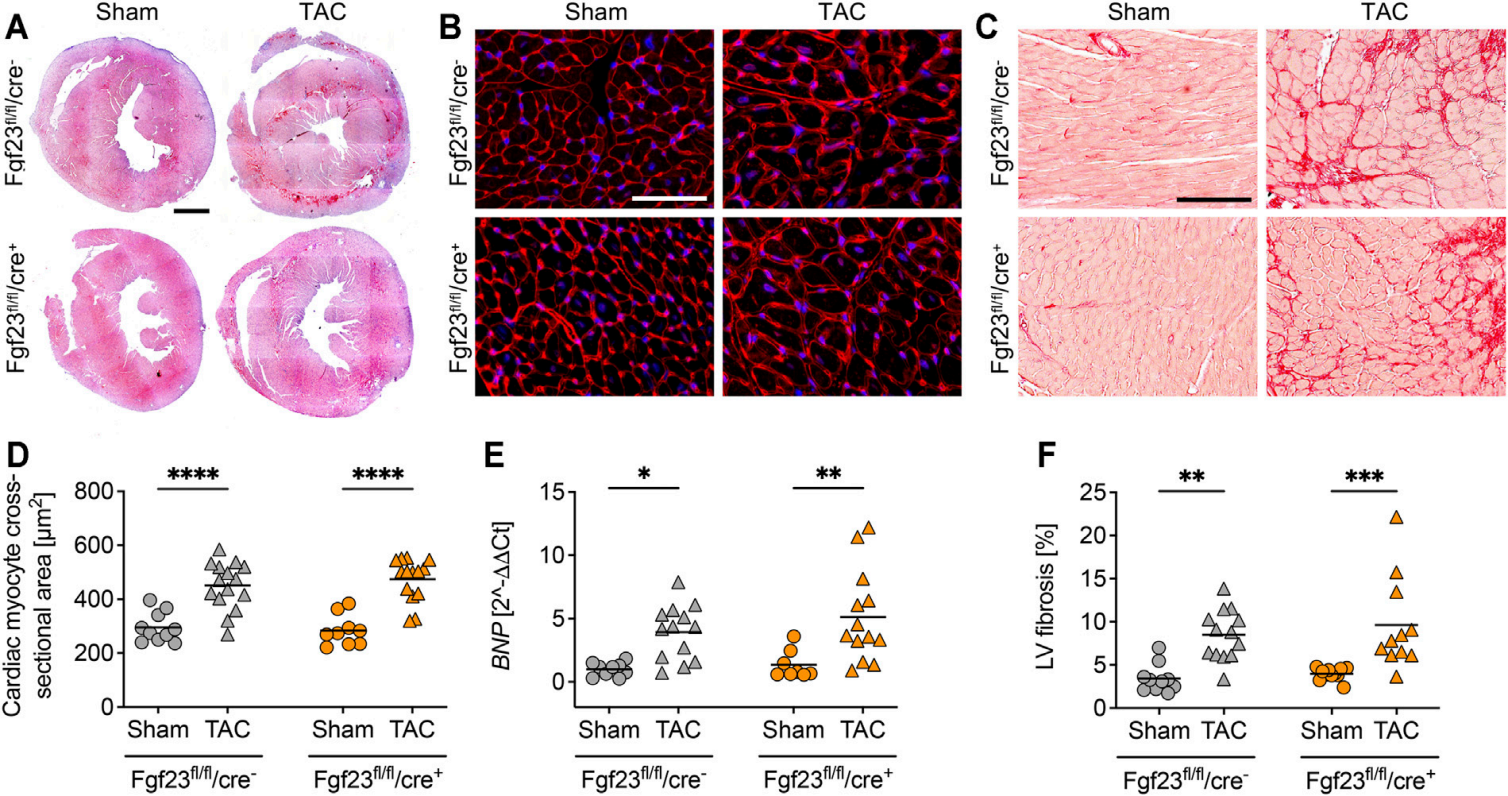
Cardiac hypertrophy and left ventricular fibrosis is equally distinct in Fgf23^fl/fl^/cre^−^ and Fgf23^fl/fl^/cre^+^ mice. **(A)** Representative heart cross-sections of sham and TAC-operated Fgf23^fl/fl^/cre^−^ and Fgf23^fl/fl^/cre^+^ mice stained with hematoxylin and eosin (H and E). Magnification ×10, scale bar 500 µm. **(B)** Representative cardiac cross-sections of sham and TAC-operated Fgf23^fl/fl^/cre^−^ and Fgf23^fl/fl^/cre^+^ mice stained with wheat germ agglutinin (WGA) Alexa Fluor 555 (red) and DAPI for nuclei (blue). Magnification ×10, scale bar 50 µm. **(C)** TAC increases cardiac myocyte cross-sectional area in both genotypes. **(D)** Cardiac mRNA expression levels of *BNP* are induced by TAC in Fgf23^fl/fl^/cre^−^ and Fgf23^fl/fl^/cre^+^ mice. **(E)** Representative images of picrosirius red-stained cardiac mid-chamber free-wall of sham and TAC-operated Fgf23^fl/fl^/cre^−^ and Fgf23^fl/fl^/cre^+^ mice. Magnification ×10, scale bar 100 µm. **(F)** Quantification of collagen fibers reveals increased left ventricular (LV) fibrosis in both Fgf23^fl/fl^/cre^−^ and Fgf23^fl/fl^/cre^+^ mice after TAC. Data is given as scatter dot plots with means; *p < 0.05, **p < 0.01, ***p < 0.001, and ****p < 0.0001 analyzed by Two-way ANOVA followed by Tukey’s multiple comparison test; *n* = 8–15 mice per group.

Enhanced circulating and cardiac FGF23 levels are shown to be associated with LVH in CKD (Faul et al., 2011; Leifheit-Nestler et al., 2016). Furthermore, high plasma FGF23 is present in patients with infarction-related cardiogenic shock and acute decompensated heart failure, which is associated with disease severity and mortality (Pöss et al., 2013; Fuernau et al., 2014; Andersen et al., 2016). It was previously shown that TAC, a model for high blood pressure-induced cardiac hypertrophy and heart failure, induces the expression of cardiac *Fgf23* in mice (Slavic et al., 2017). Thus, we next investigated whether the deletion of cardiac myocyte-specific Fgf23 is protective against pathological cardiac remodeling after aortic banding. Two weeks after TAC, cardiac function and geometry were determined by magnetic resonance imaging (MRI) (Figure 5A). TAC significantly enhanced LV mass and impaired ejection fraction (EF) in both genotypes (Figures 5B,C). It increased ESV, decreased stroke volume (SV) and enlarged systolic LV diameter in both groups (Figures 5D–F). Compared to respective TAC Fgf23^fl/fl^/cre^−^ controls, heart function seemed to be even more impaired in TAC-operated Fgf23^fl/fl^/cre^+^ mice. However, H&E-stained cardiac mid chamber cross-sections revealed increased LV wall thickness after TAC in both groups (Figure 6A). Concomitantly, similar increases in cardiac myocyte size and pro-hypertrophic *BNP* mRNA expression were seen in Fgf23^fl/fl^/cre^+^ mice and controls after TAC (Figures 6B–D). The mean percentage of LV fibrosis after TAC was similar in Fgf23^fl/fl^/cre^+^ and Fgf23^fl/fl^/cre^−^ mice (Figures 6E,F). Taken together, our findings suggest that specific disruption of *Fgf23* in cardiac myocytes does not essentially alter cardiac dysfunction in the TAC mouse model, with the exception of a modest alteration in ESV that however, does not translate to any changes in hypertrophy, fibrosis or chamber remodeling.

### Transverse Aortic Constriction Induces the Expression of Fgf23 in Cardiac Fibroblasts and Endothelial Cells

**FIGURE 7:**
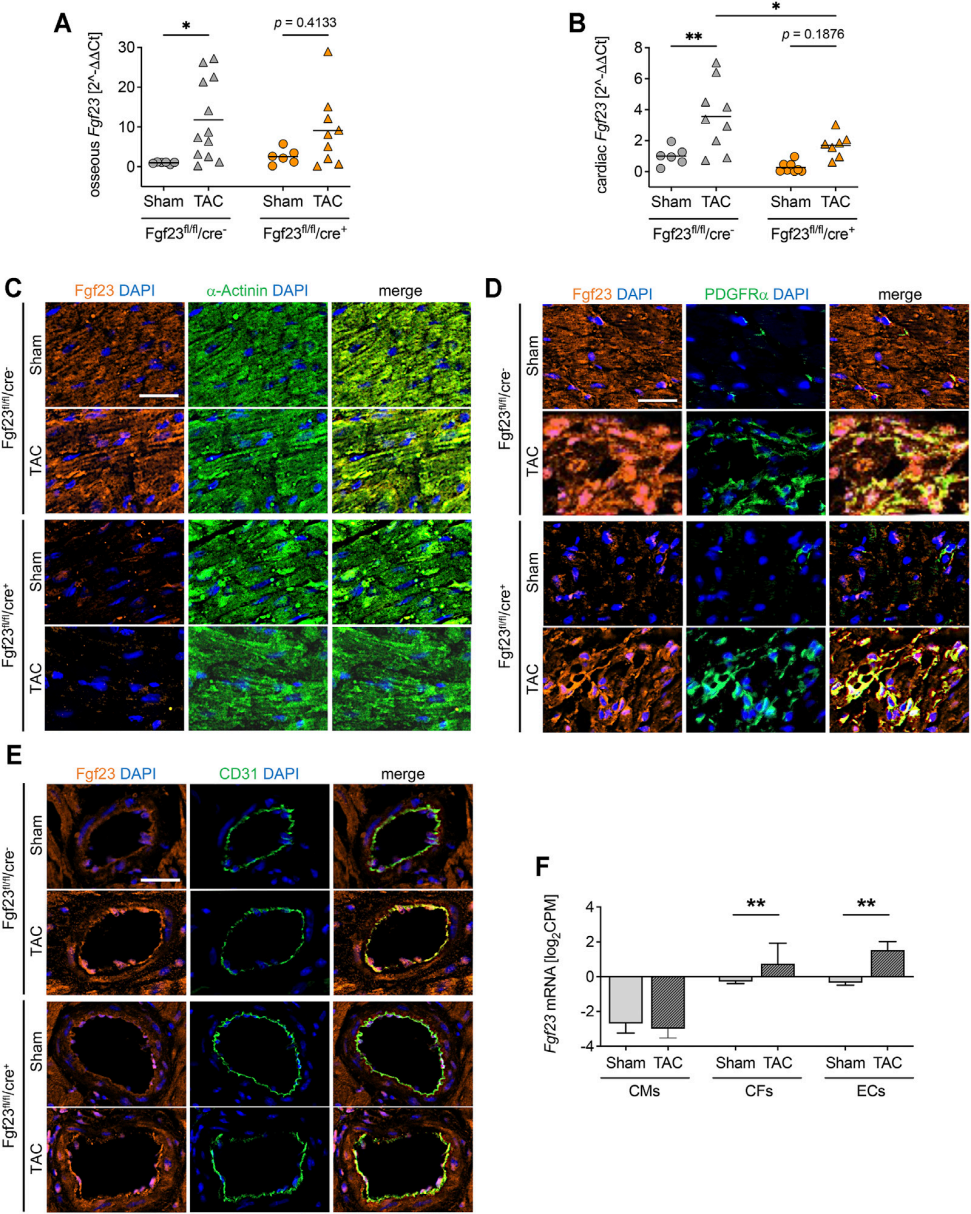
High blood pressure induces Fgf23 synthesis in cardiac fibroblasts and endothelial cells. **(A, B)** Osseous and cardiac *Fgf23* mRNA expression is increased in TAC-operated Fgf23^fl/fl^/cre^−^ and Fgf23^fl/fl^/cre^+^ mice, although cardiac Fgf23 levels are lower in Fgf23^fl/fl^/cre^+^ mice compared to Fgf23^fl/fl^/cre^−^ controls. Data is given as scatter dot plots with means; *p < 0.05 and **p < 0.01 analyzed by Two-way ANOVA followed by Tukey’s multiple comparison test; *n* = 6–12 mice per group. **(C–D)** Co-immunofluorescence images of sham and TAC-operated Fgf23^fl/fl^/cre^−^ and Fgf23^fl/fl^/cre^+^ mice stained for Fgf23 (orange) with DAPI for nuclei (blue) and **(C)** α-Actinin, **(D)** PDGFRα or **(E)** CD31 (each in green). Magnification ×20, scale bar 25 µm. **(F)** Cardiac cell type specific isolation followed by RNA-sequencing analysis from isolated cardiac myocytes (CMs), cardiac fibroblasts (CFs) and endothelial cells (ECs) after TAC or sham surgery. CPM indicates counts per million reads. Data is given as mean ± SEM of *n* = 3 cell isolations per group; **p < 0.01 analyzed by two-tailed Student’s *t*-test.

Next, we assessed the TAC-induced changes in Fgf23 synthesis. As shown by qPCR analysis, TAC stimulated the expression of *Fgf23* in bone of both Fgf23^fl/fl^/cre^+^ mice and Fgf23^fl/fl^/cre^−^ mice, although the latter did not reach the level of statistical significance in Two-way ANOVA comparisons (Figure 7A). TAC further resulted in increased *Fgf23* mRNA levels in Fgf23^fl/fl^/cre^−^ mice using total cardiac tissue (Figure 7B). Interestingly, cardiac *Fgf23* was also slightly increased after TAC in Fgf23^fl/fl^/cre^+^ mice compared to sham, although *Fgf23* mRNA levels were significantly lower than in TAC-operated controls. To elucidate the origin of TAC-induced cardiac *Fgf23*, we next performed co-immunofluorescence staining of Fgf23 with α-Actinin, PDGFRα, and CD31. TAC did not affect the synthesis of Fgf23 in cardiac myocytes (Figure 7C), while PDGFRα co-staining revealed enhanced accumulation of cardiac fibroblasts clearly expressing Fgf23 in both genotypes (Figure 7D), which was in line with severe fibrosis due to TAC as detected by picrosirius red staining (Figures 6E,F). Interestingly, TAC caused an enhanced Fgf23 synthesis in endothelial cells in both genotypes, as shown by increased Fgf23 detection in CD31-positive blood vessels (Figure 7E). The isolation of cardiac myocytes, cardiac fibroblasts and endothelial cells confirmed a specific induction of *Fgf23* transcription in cardiac fibroblasts and endothelial cells after TAC compared to sham, while the absolute *Fgf23* levels in cardiac myocytes remained unchanged (Figure 7F). Taken together, irrespective of the disruption of cardiac myocyte-specific Fgf23, TAC leads to increased cardiac Fgf23 levels that most likely results from a specific induction of Fgf23 synthesis in cardiac fibroblasts and endothelial cells.

## Discussion

Enhanced FGF23 levels are associated with the development of LVH in patients with CKD (Kovesdy and Quarles, 2016) and it has been shown that FGF23 directly induces cardiac hypertrophy *in vitro* and *in vivo* (Faul et al., 2011). Here we showed that the healthy heart is a source of Fgf23, where cardiac myocytes, fibroblasts and endothelial cells express Fgf23. Although, a cardiac myocyte-specific *Fgf23* knockout in mice did neither resulted in altered mineral metabolism, nor in cardiac dysfunction or cellular pathologies, overall mortality was enhanced in Fgf23^fl/fl^/cre^+^ mice within six months of age. After TAC, cardiac function deteriorated in Fgf23^fl/fl^/cre^+^ and Fgf23^fl/fl^/cre^−^ mice compared to sham controls and the evaluation of cardiac Fgf23 synthesis revealed elevated expression in cardiac fibroblasts and endothelial cells after TAC even in Fgf23^fl/fl^/cre^+^ mice. Interestingly, cardiac myocyte-derived Fgf23 was unaffected by TAC.

High intra-cardiac synthesis of FGF23 has been shown in patients with CKD and LVH (Leifheit-Nestler et al., 2016) as well as in animal models of experimental uremia (Leifheit-Nestler et al., 2017), myocardial infarction (Andrukhova et al., 2015; Schumacher et al., 2019) and after pulmonary artery banding (Kuga et al., 2020). It is well established that FGF23 induces LVH (Faul et al., 2011), and we and others have shown that cardiac myocytes express FGF23 *in vitro* and in human heart samples (Richter et al., 2015; Leifheit-Nestler et al., 2016; Leifheit-Nestler and Haffner, 2018). Indeed, most investigations regarding cardiac FGF23 focused on the LV and associated disorders, but here we observed that also the right ventricle is a source of Fgf23 in healthy mice. This was supported by a recently published study of Kuga et al. showing that Fgf23 promoted cardiac fibrosis in the right ventricle, predominantly mediated by the induction in cardiac myocytes (Kuga et al., 2020). FGF23 was further shown to be expressed in human coronary arteries of patients with kidney function impairment (Van Venrooij et al., 2014) underscoring our findings on the expression of Fgf23 in healthy mouse endothelium in the present study. However, the regulation of endogenous FGF23 synthesis in cardiac endothelial cells has not been addressed so far. The expression of FGF23 in cardiac fibroblasts is controversial. Here, we showed a clear expression of Fgf23 in cardiac fibroblasts of healthy mice, whereas Schumacher et al. found Fgf23 to be expressed by cardiac fibroblasts only immediately after myocardial infarction (MI) during the inflammatory phase, but neither in later phases nor in health (Schumacher et al., 2019). Hao et al. were able to detect Fgf23 expression in untreated adult mouse cardiac fibroblasts (AMCF) as well as in neonatal rat cardiac fibroblasts (NRCF) *in vitro* (Hao et al., 2016), whereas others could not detect Fgf23 in NRCF (Leifheit-Nestler et al., 2018). However, Fgf23 was also detected in kidney fibroblasts, especially myofibroblasts (Smith et al., 2017). Additionally, Smith et al. pointed out that Fgf23 was induced in kidney fibroblasts after injury, which appears to be in line with the findings of Schumacher et al. regarding the induction of Fgf23 in cardiac fibroblasts after MI (Schumacher et al., 2019) and our finding of increased Fgf23 expression in cardiac fibroblasts after TAC.

Whether different diseases stimulate cardiac FGF23 synthesis or *vice versa* high cardiac FGF23 promotes these cardiac pathologies is still not clear and the specific cellular source of cardiac FGF23 in these settings has not been sufficiently investigated. Genetically modified animal models are important to study and understand the function of specific genes in health and disease. Hereby, mouse models with deletion or overexpression of Fgf23 were critical for understanding the role of Fgf23 in the bone-kidney signaling axis (Shimada et al., 2004a and Shimada et al., 2004b). At birth, transgenic Fgf23 overexpressing mice and global Fgf23 knockout mice are phenotypically not different from wild-type mice, but develop growth retardation due to skeletal malformations at weaning age or by 13 days of age, respectively. Although both genotypes are viable, global Fgf23 knockout mice did not survive longer than 13 weeks of age (Shimada et al., 2004b). Therefore, we generated a mouse model with conditional cardiac myocyte-specific Fgf23 knockout to investigate the role of cardiac FGF23 in health and disease. We confirmed that the cardiac mRNA expression of *Fgf23* in Fgf23^fl/fl^/cre^+^ was almost undetectable and histological evaluation revealed no synthesis in cardiac myocytes. Fgf23 protein levels in whole heart tissue lysates were reduced compared to controls but still present. This could be due to unchanged Fgf23 concentrations in cardiac fibroblasts and endothelial cells. Compared to the global Fgf23 knockout mouse, Fgf23^fl/fl^/cre^+^ mice were also viable but did not show any growth retardation at birth or in later stages of life, which can be attributed to the normal bone and mineral metabolism. Heart geometry and function was not altered in mice with conditional cardiac myocyte-specific *Fgf23* disruption up to six months of age. Comparably, Fgf23^fl/fl^/cre^+^ mice showed no structural or molecular cardiac abnormalities.

Clinical studies regarding the association of high FGF23 and hypertension are controversial (Li et al., 2018; Ramezanzade et al., 2019; Drew et al., 2020). Nevertheless, *in vivo* studies showed that Fgf23 increased sodium uptake in the distal tubule resulting in volume expansion, hypertension and finally cardiac hypertrophy (Andrukhova et al., 2014). In addition, TAC-induced LVH in mice was associated with increased cardiac Fgf23 in whole tissue samples (Slavic et al., 2017; Matsui et al., 2018). Slavic et al. performed TAC in a global Fgf23 knockout mouse crossed with mice expressing a non-functioning vitamin D receptor (VDR) to prohibit vitamin D intoxication on a rescue diet to ensure normal growth, and showed no differences in the progression of cardiac hypertrophy compared to controls (Slavic et al., 2017). However, effects of non-functioning VDR or rescue diet cannot be excluded in this model and do not refute a possible causal relationship of cardiac FGF23 and pressure-induced LVH. Taken all these into account, it cannot be ruled out that loss of FGF23 in cardiac myocytes protects them from hypertrophic growth or at least reduces pro-hypertrophic signaling. To analyze whether cardiac FGF23 plays a role in pressure-induced LVH, we performed TAC on Fgf23^fl/fl^/cre^+^ and control mice. In contrast to Slavic et al., TAC even tended to worsen cardiac function in Fgf23^fl/fl^/cre^+^, although this was not translated to any alterations in cellular hypertrophy, fibrosis or chamber remodeling. Analyzing cardiac Fgf23 in more detail, our data observed increased *Fgf23* mRNA expression due to TAC even in total heart tissue of cardiac myocyte-specific Fgf23 knockout mice. Immunofluorescence staining of cardiac mid-chamber cross-sections and deep-sequencing data of isolated adult cardiac myocytes, cardiac fibroblasts and endothelial cells revealed a specific induction of Fgf23 synthesis in cardiac fibroblasts and endothelial cells after TAC, which explain enhanced Fgf23 expression even in TAC-operated hearts of Fgf23^fl/fl^/cre^+^ mice. Taken all this into account, cardiac myocyte specific depletion of Fgf23 might not have a great impact in heart performance and structure at both basal and after stressing conditions due to the expression in other cardiac cell types. Additionally, enhanced expression and secretion of cardiac fibroblast-derived Fgf23 may stimulate myofibroblast differentiation and promote hypertrophic growth of cardiac myocytes in an endocrine manner even in Fgf23^fl/fl^/cre^+^ mice after TAC. The deleterious effect of high Fgf23 on the endothelium has also been discussed before (Six et al., 2014; Silswal N et al., 2014; Verkaik et al., 2018; Richter et al., 2016). Thus, Fgf23 secreted from cardiac fibroblast may further affect endothelial cells in Fgf23^fl/fl^/cre^+^ mice in a paracrine manner that are already under stress due to high pressure after TAC. Crosstalk between endothelial cells and cardiac myocytes is also known and intensively studied (Chen et al., 2010; Zhang et al., 2015; Kivelä et al., 2019). Here, we showed that Fgf23 synthesis was induced in endothelial cells in Fgf23^fl/fl^/cre^+^ and control mice after TAC compared to respective sham-operated animals, which could further strengthen cardiac myocytes hypertrophy irrespective of cardiac myocyte-derived Fgf23.

Taken together, we showed that besides the LV, FGF23 is also expressed in the right ventricle and thus, beside LVH, could play a role in other cardiac diseases. Our data further point out that FGF23 is not only expressed in cardiac myocytes but also in cardiac fibroblasts and endothelial cells and thereby may impact heart development, and physiological and pathological cardiac function. Additionally, we investigated for the first-time cell type-specific regulation of FGF23 in heart tissue due to high blood pressure showing increased synthesis in cardiac fibroblasts and endothelial cells. Exact molecular mechanisms and the crosstalk between cardiac myocytes and non-cardiac myocytes with respect to FGF23 and its impact for cardiac physiology and pathology have to be addressed in respective *in vivo* and *in vitro* settings in further studies.

## Data Availability

The original contributions presented in the study are included in the article/Supplementary Material. Further inquiries can be directed to the corresponding author.
