# Supplementary material for: Comprehensive Expression Analysis of Cardiac Fibroblast Growth Factor 23 in Health and Pressure-induced Cardiac Hypertrophy
Source: Front Cell Dev Biol. 2022 Jan 18;9:791479. doi: 10.3389/fcell.2021.791479 (PMC8804498; doi:10.3389/fcell.2021.791479)
Supplement: Supplementary file 1 [file DataSheet1.PDF]

Supplementary Material

SUPPLEMENTARY TABLE 1. Murine primer sequences for qRT-PCR analysis.

Gene	Orientation	Sequence (5`-3`)
18S	forward	TTC TGG CCA ACG GTC TAG ACA AC
	reverse	CCA GTG GTC TTG GTG TGC TGA
ANP	forward	ATT GAC AGG ATT GGA GCC CAG AG
	reverse	TGA CAC ACC ACA AGG GCT TAG GA
BNP	forward	GAG GTC ACT CCT ATC CTC TGG
	reverse	GCC ATT TCC TCC GAC TTT TCT C
Col1a1	forward	CCG CTG GTC AAG ATG GTC
	reverse	CCT CGC TCT CCA GCC TTT
Fgf23	forward	TCA TGG CTC CTG TTA TCA CC
	reverse	GGA CCA GCT ATC ACC TAC AGA
Gapdh	forward	TAT GTC GTG GAG TCT ACT GG
	reverse	AGT GAT GGA TGG ACT GTG G

SUPPLEMENTARY TABLE 2. Blood and urine biochemistry of one to six months old Fgf23$^{fl/fl}$/cre$^-$ and Fgf23$^{fl/fl}$/cre$^+$ mice.

	1 month		3 months		6 months	
**Parameter**	**Fgf23$^{fl/fl}$/cre$^-$**	**Fgf23$^{fl/fl}$/cre$^+$**	**Fgf23$^{fl/fl}$/cre$^-$**	**Fgf23$^{fl/fl}$/cre$^+$**	**Fgf23$^{fl/fl}$/cre$^-$**	**Fgf23$^{fl/fl}$/cre$^+$**
Total Fgf23 [pg/mL]	299 [266, 425]	431 [377, 580]	321 [285, 360]	316 [198, 474]	689 [496, 1229]	521 [456, 1418]
iFgf23 [pg/mL]	272 [222, 287]	330 [271, 362]	325 [318, 396]	351 [304, 405]	378 [286, 476]	313 [287, 418]
S-phosphate [mg/dL]	8.74 ± 0.46	9.15 ± 0.54	6.88 ± 1.15	6.77 ± 0.45	7.97 ± 1.92	7.95 ± 1.16
TRP [%]	-	-	94 [90, 98]	93 [87, 94]	95 [82, 98]	93 [83, 99]
S-calcium [mg/dL]	10.6 [9.9, 11.0]	10.6 [10.2, 10.8]	9.7 [8.0, 10.6]	8.9 [7.9, 9.9]	9.6 [8.9, 10.6]	10.4 [8.7, 10.5]

Values are presented as mean ± SD, or median [interquartile range] of each n = 5-6 mice per group. Statistics are performed using Two-way ANOVA followed by Tukey´s multiple comparison test. iFgf23, intact fibroblast growth factor 23; S, serum; TRP, tubular reabsorption of phosphate.

SUPPLEMENTARY TABLE 3. Age-dependent body measurements of one to six months old $Fgf23^{fl/fl}/cre^-$ and $Fgf23^{fl/fl}/cre^+$ mice.

	**1 month**		**3 months**		**6 months**	
**Parameter**	**$Fgf23^{fl/fl}/cre^-$**	**$Fgf23^{fl/fl}/cre^+$**	**$Fgf23^{fl/fl}/cre^-$**	**$Fgf23^{fl/fl}/cre^+$**	**$Fgf23^{fl/fl}/cre^-$**	**$Fgf23^{fl/fl}/cre^+$**
Body weight [g]	14.4 ± 1.1	14.4 ± 1.6	21.1 ± 1.5[a]	21.2 ± 2.2[a]	29.8 ± 2.9[a,b]	28.0 ± 5.6[a,b]
Body length [mm]	148 [144, 152]	150 [146, 155]	170 [165, 175][a]	172 [171, 180][a]	180 [174, 192][a,b]	184 [172, 195][a]
Tibia length [mm]	14.7 [14.0, 16.6]	14.8 [14.2, 16.2]	18.0 [17.0, 18.9][a]	18.0 [17.0, 18.6][a]	19.5 [18.3, 20.0][a]	20.0 [19.5, 20.6][a,b]
Rel. HW [mg/mm]	4.9 ± 0.5	4.9 ± 0.6	5.8 ± 0.7	5.8 ± 0.9	7.7 ± 1.2[a,b]	7.5 ± 2.1[a,b]
Rel. LW [mg/mm]	6.9 ± 0.8	7.4 ± 0.9	7.2 ± 1.0	6.5 ± 1.4	7.9 ± 0.5	8.5 ± 1.8
Rel. KW [mg/mm]	11.9 ± 0.7	12.6 ± 1.7	13.5 ± 1.4	14.7 ± 2.4	18.3 ± 4.1[a,b]	16.9 ± 3.1[a]
Rel. LVW [mg/mm]	58.0 [54.6, 62.2]	55.9 [42.9, 61.0]	67.6 [54.7, 74.1]	63.4 [60.0, 71.0][a]	70.0 [60.8, 73.6]	69.9 [58.9, 75.4][a]

Values are presented as mean ± SD, or median [interquartile range] of each n = 7-11 mice per group. $^{a}p < 0.05$ compared to 1 month old mice of the same genotype, and $^{b}p < 0.05$ compared to 3 months old mice of the same genotype using Two-way ANOVA followed by Tukey´s multiple comparison test. BW, body weight; HW, heart weight; LW, lung weight; KW, kidney weight; LVW, liver weight

SUPPLEMENTARY TABLE 4. Parameters of cardiac function and geometry of one to six months old Fgf23$^{fl/fl}$/cre^{-} and Fgf23$^{fl/fl}$/cre^{+} mice determined by echocardiography.

	**1 month**		**3 months**		**6 months**	
**Parameter**	**Fgf23$^{fl/fl}$/cre^{-}**	**Fgf23$^{fl/fl}$/cre^{+}**	**Fgf23$^{fl/fl}$/cre-**	**Fgf23$^{fl/fl}$/cre^{+}**	**Fgf23f$^{l/fl}$/cre^{-}**	**Fgf23$^{fl/fl}$/cre^{+}**
LV mass [mg]	66.4 ± 6.2	65.1 ± 14.0	73.6 ± 13.0	78.1 ± 10.0	96.4 ± 21.2	80.6 ± 32.2
EF [%]	80 [73, 83]	74 [72, 81]	66 [64, 75]	63 [55, 81]	45 [36, 57]^{a,b}	39 [33, 67]^{a}
FS [%]	47 ± 5	43 ± 4	38 ± 8	38 ± 13	42 ± 15	48 ± 13
ESV [µl]	9.2 ± 2.5	10.0 ± 4.8	17.1 ± 8.8	16.7 ± 7.4	34.2 ± 14.4^{a,b}	22.5 ± 7.7^{c}
EDV [µl]	43.5 ±8.0	41.6 ± 11.0	50.0 ± 14.5	49.8 ± 11.0	70.4 ± 17.2^{a}	55.6 ± 15.7
LVPWs [µm]	1.20 [1.04, 1.33]	1.09 [0.96, 1.35]	0.99 [0.87, 1.07]	1.13 [1.05, 1.37]	0.88 [0.73, 0.97]	0.9 [0.74, 1.33]
LVPWd [µm]	0.86 [0.68, 0.87]	0.66 [0.58, 0.89]	0.73 [0.6, 0.96]	0.92 [0.69, 0.99]	0.72 [0.56, 0.8]	0.73 [0.55, 1.06]
IVSs [µm]	1.06 ± 0.14	1.03 ± 0.19	1.05 ± 0.25	1.06 ± 0.15	1.15 ± 0.19	1.12 ± 0.21
IVSd [µm]	0.80 ± 0.07	0.85 ± 0.15	0.83 ± 0.16	0.83 ± 0.13	0.92 ± 0.11	0.84 ± 0.16
LVDs [mm]	1.77 [1.56, 1.92]	1.92 [1.55, 1.99]	2.11 [2.09, 2.42]	2.31 [1.78, 2.48]	3.22 [2.25, 3.25]^{a}	2.60 [2.11, 2.77]^{a}
LVDd [mm]	3.27 ± 0.24	3.19 ± 0.39	3.44 ± 0.46	3.46 ± 0.31	3.98 ± 0.43	3.60 ± 0.47

Values are presented as mean ± SD, or median [interquartile range] of each *n* = 4-10 mice per group. $^{a}p < 0.05$ compared to 1 month old mice of the same genotype, $^{b}p < 0.05$ compared to 3 months old mice of the same genotype, and $^{c}p < 0.05$ compared to Fgf23$^{fl/fl}$/cre^{-} of the same age using Two-way ANOVA followed by Tukey´s multiple comparison test. LV, left ventricular; ES, ejection fraction; FS, fraction shortening; ESV, end-systolic volume; EDV, end-diastolic volume; s, systolic; d, diastolic; LVPW, left ventricular posterior wall thickness; IVS, intraventricular septum thickness; LVD, left ventricular diameter

SUPPLEMENTARY FIGURE 1

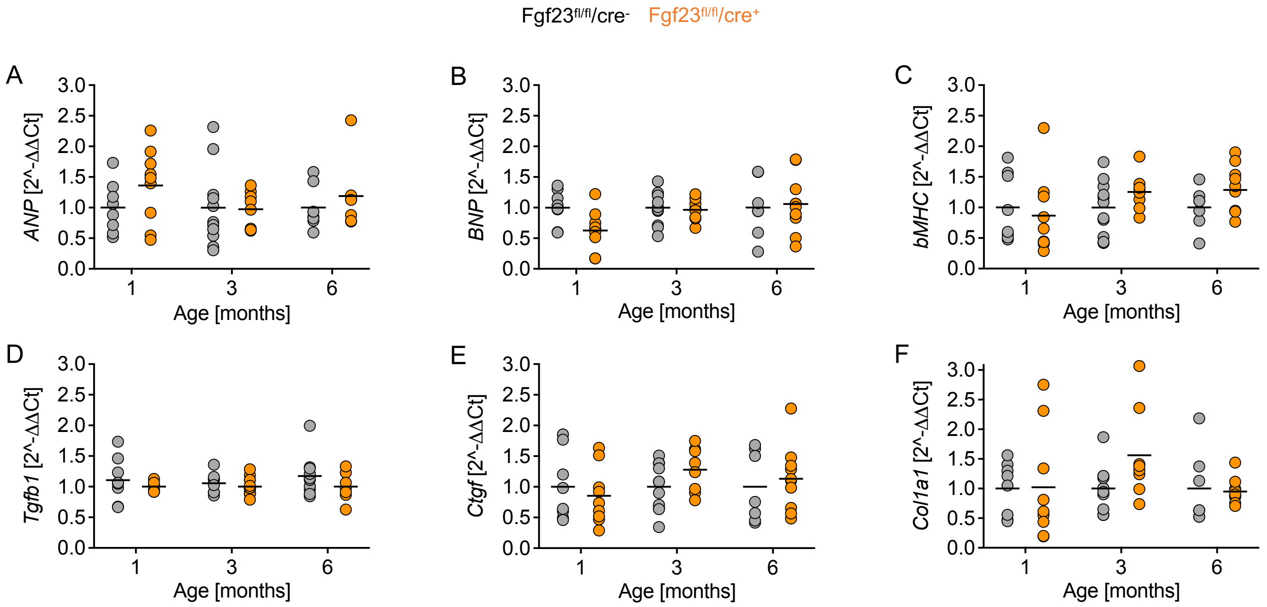

Supplementary Figure 1. Pro-hypertrophic and pro-fibrotic genes are not altered in cardiac myocyte-specific *Fgf23* knockout mice. **(A-C)** Quantitative real-time PCR analysis of atrial natriuretic peptide (*ANP*), brain natriuretic peptide (*BNP*) and beta myosin heavy chain (*bMHC*) in Fgf23$^{fl/fl}$/cre^{+} and control mice demonstrates any alterations irrespective of age. **(D-F)** Using quantitative real-time PCR analysis, the mRNA expression of transforming growth factor beta 1 (*Tgfb1*), connective tissue growth factor (*Ctgf*) and collagen type 1, alpha 1 (*Col1a1*) over time is equal between both groups. Data is given as scatter dot plots with means analyzed by Two-way ANOVA followed by Tukey´s multiple comparison test; *n* = 5-9 mice per group.
